# *Tnni3k* Modifies Disease Progression in Murine Models of Cardiomyopathy

**DOI:** 10.1371/journal.pgen.1000647

**Published:** 2009-09-18

**Authors:** Ferrin C. Wheeler, Hao Tang, Odessa A. Marks, Tracy N. Hadnott, Pei-Lun Chu, Lan Mao, Howard A. Rockman, Douglas A. Marchuk

**Affiliations:** 1 Department of Molecular Genetics and Microbiology, Duke University, Durham, North Carolina, United States of America; 2 Department of Medicine, Duke University, Durham, North Carolina, United States of America; Harvard Medical School, United States of America

## Abstract

The Calsequestrin (*Csq*) transgenic mouse model of cardiomyopathy exhibits wide variation in phenotypic progression dependent on genetic background. Seven heart failure modifier (*Hrtfm*) loci modify disease progression and outcome. Here we report *Tnni3k* (*cardiac Troponin I-interacting kinase*) as the gene underlying *Hrtfm2*. Strains with the more susceptible phenotype exhibit high transcript levels while less susceptible strains show dramatically reduced transcript levels. This decrease is caused by an intronic SNP in low-transcript strains that activates a cryptic splice site leading to a frameshifted transcript, followed by nonsense-mediated decay of message and an absence of detectable protein. A transgenic animal overexpressing human *TNNI3K* alone exhibits no cardiac phenotype. However, *TNNI3K*/*Csq* double transgenics display severely impaired systolic function and reduced survival, indicating that *TNNI3K* expression modifies disease progression. TNNI3K expression also accelerates disease progression in a pressure-overload model of heart failure. These combined data demonstrate that *Tnni3k* plays a critical role in the modulation of different forms of heart disease, and this protein may provide a novel target for therapeutic intervention.

## Author Summary

Heart failure is the common final outcome of many forms of acute and chronic heart disease. The prognosis of heart disease is highly variable between patients, and these differences in the phenotypic expression (symptoms, course, and final outcome) are in part due to genetic factors that have proven difficult to directly identify in the human population. To overcome this limitation, we employed a disease-sensitized mouse model of dilated cardiomyopathy to identify genes that modify the progression and outcome of the phenotype. Here we report the identification of a novel heart disease modifier gene, *Tnni3k*, that accelerates disease progression in two distinct mouse models of cardiomyopathy. This gene appears to play a critical role in modulating heart disease phenotypes and may provide a novel target for therapeutic intervention.

## Introduction

Heart failure is the common final outcome for many forms of acute and chronic heart disease. The prognosis of heart failure is highly variable between patients, and the differences in phenotypic expression (symptoms, disease progression and course, and final outcome) create difficulties in the construction of predictive models [1]. Previous research has suggested that genetic factors can considerably modify the progression and outcome of heart failure [2]. However, these factors are difficult to identify directly in the human population because of wide genetic variability, uncontrollable environmental factors, and the intervention of medical therapy.

We have employed a disease-sensitized mouse model to map genetic factors that modify the progression and outcome of heart disease. In the Calsequestrin transgenic mouse, cardiac-specific overexpression of the calcium binding protein Calsequestrin (CSQ) leads to dilated cardiomyopathy [3]. This murine model recapitulates many of the hallmarks of human dilated cardiomyopathy including cardiac enlargement, depressed contractile function, abnormal beta-adrenergic receptor signaling and premature death [4]. Although all mice that overexpress CSQ develop dilated cardiomyopathy, disease progression and outcome varies greatly depending on the genetic background (inbred mouse strain) harboring the *Csq* transgene (*Csq^tg^*) [5],[6]. These differences are due to modifier genes whose multiple alleles differentially modulate the phenotype.

We have exploited these strain-specific phenotypic differences to map seven different loci (Heart failure modifier or *Hrtfm*) that modify the progression of cardiac dysfunction and the outcome of heart failure [5]–[7]. *Hrtfm2*, mapping to mouse chromosome 3, was identified in a cross between inbred strain DBA/2J (DBA), which harbors the original *Csq^tg^*, and C57BL/6 (B6). In this cross, the B6 allele at *Hrtfm2* imparted a dominant, disease-accelerating effect on both cardiac dysfunction (as measured by echocardiography) and reduced survival [5]. Subsequently, *Hrtfm2* was re-identified in a second cross between DBA/*Csq^tg^* animals and inbred strain AKR/J (AKR) [7]. In this cross, the AKR allele of *Hrtfm2* also imparted a dominant, disease-accelerating effect on cardiac dysfunction and survival. The phenotypic effects of *Hrtfm2* were robust, accounting for 30% of the genetic variability for survival and 22% for cardiac dysfunction. Capitalizing on the ancestral nature of the *Hrtfm2* allele (ie, mapping within a murine haplotype block that has been retained throughout evolution and now found in multiple inbred strains), we employed haplotype-sharing analysis to effectively narrow the candidate interval from 16.5 Mb to 2 Mb, a region containing only 7 known genes [7]. We had previously suggested the *Tnni3k* gene as an attractive candidate based on its location within the shared haplotype interval and its biological significance as a cardiac-specific kinase that reportedly interacts with cardiac Troponin I (cTnI) [8]. Here we report the molecular characterization of allelic variation at the murine *Tnni3k* gene, and present *in vivo* functional evidence showing that *Tnni3k* underlies the heart failure modifier locus, *Hrtfm2*.

## Results

**Figure 1:**
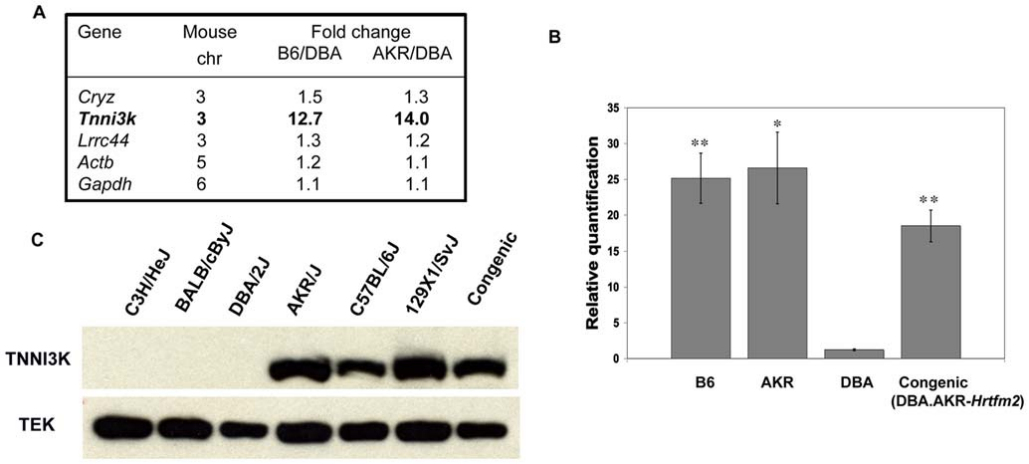
*Tnni3k* mRNA and protein expression varies significantly between mouse strains. (A) Affymetrix microarray analysis identified only one gene on murine chromosome 3 with a significant expression change between B6, AKR, and DBA. Two genes flanking *Tnni3k* (*Cryz* and *Lrrc44*) that are expressed at similar levels in all strains are shown, as well as two control genes, *Actb* (*β-actin*) and *Gapdh*. (B) qRT-PCR confirms expression differences identified by microarray analysis. TaqMan qRT-PCR of *Tnni3k* from 5 wild-type mouse hearts from each strain confirms that transcript levels are higher (approximately 25-fold) in B6 and AKR compared to DBA (**p>0.0001 and *p>0.001). Three hearts from the *Hrtfm2* congenic line harboring AKR alleles at the *Tnni3k* locus on a DBA genetic background (DBA.AKR-*Hrtfm2*) shows transcript levels similar to B6 and AKR hearts, which is significantly higher than observed in DBA hearts (**p>0.0001). *Actb* served as an endogenous control. Error bars indicate standard error of the mean (SEM). (C) Western blot analysis shows that three strains that share the DBA haplotype at *Tnni3k* show no detectable TNNI3K protein, while three strains with the B6 haplotype show robust expression. The DBA.AKR-*Hrtfm2* congenic mouse shows high expression as predicted based on RNA expression. Receptor tyrosine kinase *TEK* was used as a protein loading control.

As part of an effort to identify candidate genes for the *Hrtfm* loci, we performed microarray expression analysis of normal heart tissue from the inbred strains used in our mapping crosses to identify genes showing innate differences in transcript levels. Of the genes mapping within the *Hrtfm2* linkage peak, only one gene exhibited significantly different transcript levels between the less susceptible strain DBA and the more susceptible strains B6 and AKR. Transcript levels of *Tnni3k* were elevated 12-fold in B6 and AKR compared with DBA, whereas levels of other transcripts mapping within the interval were not significantly different (Figure 1A). These expression differences were validated by more-sensitive qRT-PCR analysis, where *Tnni3k* message levels were found to be 25-fold higher in B6 and AKR strains than those in DBA (Figure 1B). In parallel, we genetically isolated the *Hrtfm2* locus by creating a congenic line that carried AKR alleles across *Hrtfm2* (an approximately 20 Mb region between rs13477425 and rs13477504) and DBA alleles throughout the rest of the genome. Quantitative RT-PCR showed that *Tnni3k* transcript levels in hearts from DBA.AKR-*Hrtfm2* congenic mice were comparable to levels observed in B6 and AKR (AKR being the source of the *Hrtfm2* locus), and not that seen in DBA (the genomic background), suggesting that the *Tnni3k* expression differences were driven by *cis*-acting sequence elements within the *Hrtfm2* locus, rather than *trans*-acting factors mapping elsewhere in the genome.

We analyzed heart tissue prepared from six inbred mouse strains to determine if these differences in levels of *Tnni3k* transcript would be observed at the protein level. We chose three additional strains that shared either the DBA or B6 haplotype at *Tnni3k* (Table S1). As predicted by the transcript levels, robust levels of TNNI3K protein were detected in B6, AKR, 129×1/SvJ (129) and the DBA.AKR-*Hrtfm2* congenic, which share the B6 haplotype. Surprisingly, no apparent protein was detected for DBA, C3H/HeJ and BALB/cByJ (BALB/c) strains, which share the DBA haplotype (Figure 1C). Therefore, within the limits of detection of the antiserum, TNNI3K protein was apparently absent from hearts of strains sharing the DBA haplotype at the *Tnni3k* locus. The latter strains effectively represent *Tnni3k* null or extreme hypomorphic genotypes with no apparent effect on development or survival, and with no obvious pathological consequence.

**Figure 2:**
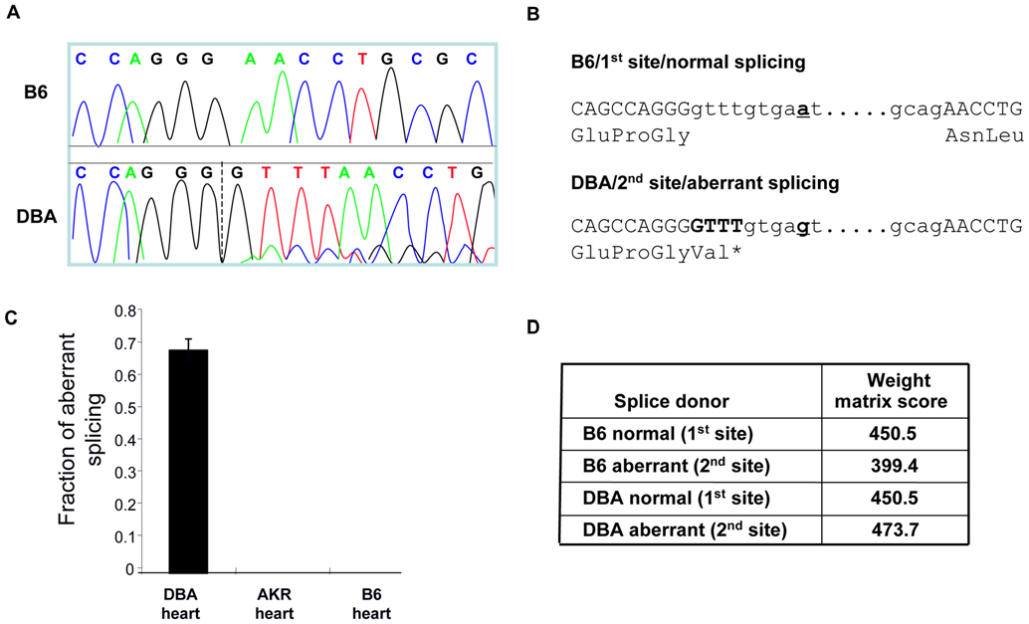
Aberrant splicing of *Tnni3k* in hearts from DBA mice. (A) Sequencing chromatogram shows the exon 19–20 boundary in *Tnni3k* cDNA from B6 and DBA hearts. The dashed line shows the first base of the 4 nucleotide cDNA insertion (GTTT) derived from intron 19. The small proportion of properly spliced transcript in DBA can be seen as overlapping sequence after the dashed line. (B) Sequence of exon 19 and 20 with flanking intronic sequence with amino acid translation for both B6 (1^st^ site/normal) and DBA (2^nd^ site/aberrant). The 4 nucleotide GTTT insertion is shown in bold, and rs49812611 is underlined. (C) Fluorescent fragment analysis was used to determine the fraction of aberrant splicing in DBA. Almost 70% of total message in DBA was aberrantly spliced, while no aberrant splicing was observed in B6 and AKR. (D) Weight matrix scores [9],[10] for the different splice donor sites are shown.

The *Tnni3k* coding region of the mapping strains differed by a single, relatively conservative, non-synonymous coding SNP (rs30712233, T659I). By sequencing *Tnni3k* cDNA from the strains, we noted another, more consequential, strain-specific sequence difference. All strains sharing the B6 haplotype showed a single major transcript identical to the published cDNA. By contrast, all strains sharing the DBA haplotype exhibited a mixture of two transcripts consisting of the published transcript and a second transcript containing a 4-nucleotide insertion between exons 19 and 20 (Figure 2A). This insertion was not present in the genomic DNA, but instead represented the addition of the first 4 nucleotides from intron 19 into the *Tnni3k* transcript. The insertion created a frameshift resulting in a premature termination codon immediately downstream (Figure 2B). We determined that the frameshifted transcript accounted for approximately 70% of the message in DBA heart mRNA, but was not present in B6 or AKR (Figure 2C). This transcript was not found in any of the EST databases for mouse or any other species, suggesting that it represented an aberrant message created by defective splicing, possibly caused by the use of a second ‘gt’ splice donor site 4 nucleotides downstream of the normal donor site.

The genomic region surrounding exons 19 and 20 harbors over 50 SNPs. Although in principle any of these could have caused the aberrant splicing, we focused on the SNP nearest to the splice donor junction. B6 and related strains (AKR, 129, MRL) possess an ‘a’ nucleotide at rs49812611, whereas DBA and related strains (A/J, C3H/HeJ (C3H), BALB/c) possess a ‘g’. This SNP lies at the +9 position for the normal splice site, but importantly, this SNP lies at the +5 position with reference to the aberrant splice site. Thus, DBA and related strains harbor the consensus ‘g’ nucleotide at the +5 position for the aberrant site. During mRNA processing, the ‘g’ at the +5 splice donor position pairs with a ‘c’ in the U1 or U6 snRNA, resulting in a preference for ‘g’ at this position. Weight matrix scores for splice donor strength [9],[10] for each possible splice donor site confirmed that the second (aberrant) splice site was the strongest splice site in the region only when the ‘g’ nucleotide is present at rs49812611 (Figure 2D).

**Figure 3:**
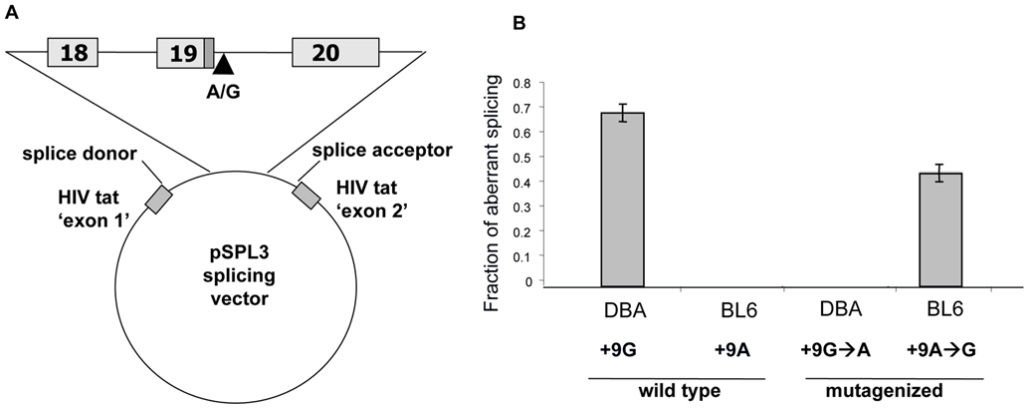
The sequence at rs49812611 is responsible for aberrant *Tnni3k* splicing. An *in vitro* system was used to test the role of the intron 19 SNP (rs49812611) in aberrant splicing between exons 19 and 20 in DBA compared to B6. (A) Schematic representation of the *Tnni3k* exon 18–20 *in vitro* splicing construct used to test aberrant splicing. Genomic fragments (4 kb) from DBA and B6 including exons 18, 19, and 20 were amplified and cloned into a splicing vector. Additionally, site-directed mutagenesis was used to alter the sequence at the SNP in both constructs. Splicing constructs were transfected into 293T cells and RNA was harvested after 48 hours. (B) Analysis of *Tnni3k* splicing reveals aberrant splicing of the *in vitro* DBA construct closely resembles splicing in wild-type DBA hearts but the aberrant transcript is absent with the B6 *in vitro* construct. When the critical nucleotide at the +9 position in intron 19 is exchanged between the constructs, the splicing pattern follows the sequence at the SNP, demonstrating that the sequence at rs49812611 is responsible for the splicing defect.

We tested the hypothesis that rs49812611 is the cause of aberrant splicing using an *in vitro* splicing system. Genomic DNA spanning exons 18 through 20 from both B6 and DBA were sub-cloned and transfected into 293T cells (Figure 3A). These *in vitro* constructs recapitulated the splicing pattern observed *in vivo*, confirming that the splicing defect was caused by *cis*-acting sequences residing within the cloned 4 kb genomic fragment (Figure 3B). Site-directed mutagenesis was used to investigate the role of rs49812611 in aberrant splicing. A single change at this SNP completely reversed the splicing pattern. DBA genomic DNA altered to carry the ‘a’ allele at rs49812611 generated no aberrant splice product, whereas the B6 DNA carrying the ‘g’ allele exhibited the aberrant product (Figure 3B). These results showed that rs49812611 was responsible for the presence or absence of the aberrantly spliced message, although the extent of aberrant splicing may be modulated by other flanking sequence variation.

**Figure 4:**
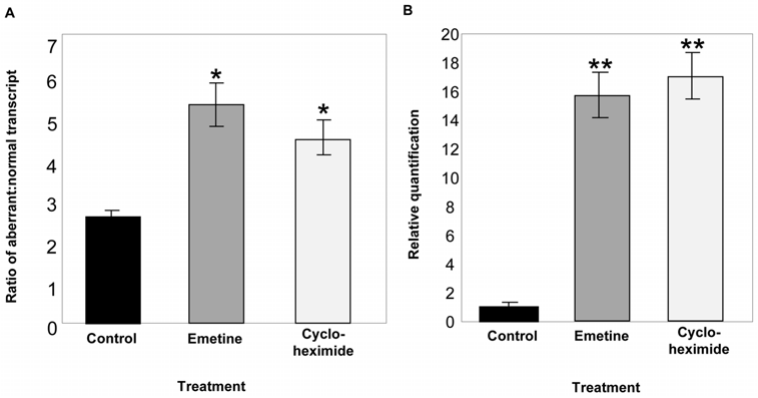
Nonsense-mediated decay is responsible for reduced *Tnni3k* transcript levels. HL-1 cardiomyocytes were treated with emetine or cycloheximide to block NMD. RNA was isolated from cells 24 hours after treatment. The ratio of aberrant to wild-type transcripts was calculated and qRT-PCR was used to determine *Tnni3k* message levels relative to *actb*. Cells that were mock-treated with the drug-diluent buffer solution acted as a control. (A) Either emetine or cycloheximide treatment preferentially increases levels of the aberrantly spliced message relative to the normally spliced message (*p<0.01). (B) Either emetine or cycloheximide treatment increased the total levels of *Tnni3k* message approximately 16-fold above mock-treated cells (**p<0.001).

Since *Tnni3k* was originally identified as a positional candidate for *Hrtfm2* due to differences in transcript levels between the mapping strains, we hypothesized that nonsense-mediated decay (NMD) was responsible for the drastically reduced levels of the frameshifted message seen in DBA. We investigated this in the mouse cardiomyocyte cell line, HL-1 [11], which shares the DBA haplotype at *Tnni3k*. We first confirmed that HL-1 cells expressed both aberrant and normal *Tnni3k* at levels comparable to wild-type DBA hearts, with the majority of the message being the aberrant variant that includes the 4-nucleotide insertion. HL-1 cardiomyocyte cells were then treated with either cycloheximide and emetine, two drugs commonly used to block NMD [12]. Treatment with either drug increased the level of aberrantly spliced transcript relative to the normally spliced message (Figure 4A). As predicted, these treatments increased levels of total *Tnni3k* mRNA 16-fold (Figure 4B), supporting a major role for NMD in the observed differences in transcript levels between strains.

Although these experiments determined the molecular mechanism underlying the observed differences in *Tnni3k* transcript levels, they did not address the *in vivo* role of *Tnni3k* in the progression of cardiomyopathy. We next investigated whether *Tnni3k* was the gene underlying the *Hrtfm2* locus. We created transgenic mouse lines that expressed human TNNI3K protein in the heart. TNNI3K protein is highly conserved between human and mouse (91% identity), and transgenic expression of the human transcript enabled discrimination between the endogenous murine transcript and that derived from the transgene. Three independent founder lines were created, and qRT-PCR indicated that the human transgene was expressed at levels ranging from 5 to 20-fold above the endogenous B6 mouse transcript, depending on the founder. F1 generation mice from all three lines survived over a year, and cardiac function in 12 and 21-week transgenic animals were indistinguishable from wild-type animals. Consequently, TNNI3K expression alone did not result in overt cardiomyopathy or decreased survival due to heart failure. This was not unexpected, since in the absence of the *Csq* transgenic disease-sensitizer, there were no measurable differences in heart function between B6 and DBA animals, even though B6 express robust levels of TNNI3K whereas DBA shows no detectable protein.

By repeated backcrosses to DBA, the *TNNI3K* transgenes were introgressed into the DBA background that shows no detectable endogenous murine TNNI3K protein to test the hypothesis that in the presence of the *Csq* transgenic sensitizer, increased expression of TNNI3K would accelerate disease progression. The backcrossed transgenic lines continued to express robust levels of the human TNNI3K protein (Figure S1). Two lines were chosen for all subsequent experimental crosses, and phenotypic data from N5 (or N6) animals from both lines were combined, as there was no discernable difference in the data derived from either transgenic line.

**Figure 5:**
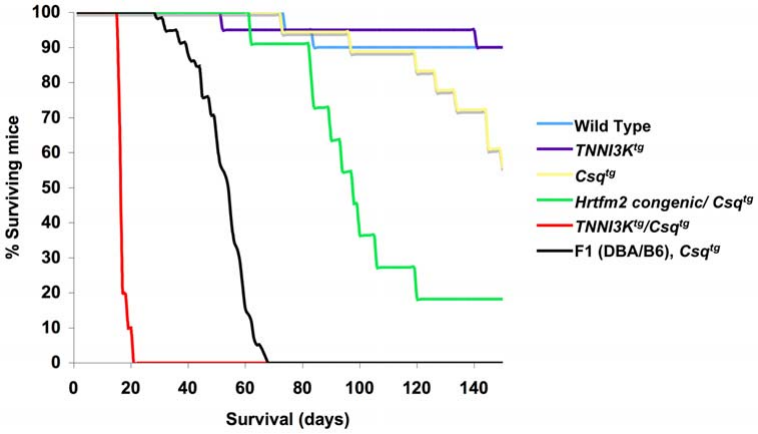
TNNI3K expression leads to premature death in the *Csq* transgenic model of cardiomyopathy. A Kaplan-Meier survival graph shows the outcomes of different genotypic groups resulting from a cross between *TNNI3K^tg^* and *Csq^tg^* transgenic animals. Survival is severely decreased for double positive transgenics (*TNNI3K^tg^*/*Csq^tg^*) to an average of 17 days with a range from 15 to 21 days. *Csq^tg^* mice on congenic background (DBA. AKR-*Hrtfm2* : DBA genome except *Hrtfm2* loci) survived to 107 days on average. Nearly all mice with other genotypes, including both single positives (*TNNI3K^tg^* and *Csq^tg^*) survived to at least 150 days, at which point the animals were sacrificed and the experiment terminated. Survival of *TNNI3K^tg^*/*Csq^tg^* and Congenic/*Csq^tg^* compared to the three other groups was significantly decreased (p<0.00001). F1(DBA/B6) *Csq^tg^* animals died much sooner (average only 50 days) when compared to *Csq^tg^* in the background of the isolated (congenic) *Hrtmf2* locus. The number of animals in each group is as follows: Wild type, n = 18; *TNNI3K^tg^*, n = 18; *Csq^tg^*, n = 18; Congenic/*Csq^tg^*, n = 11; *TNNI3K^tg^*/*Csq^tg^*, n = 12, F1(DBA/B6) *Csq^tg^*, n = 58 (F1 data from [5]).

In addition to an apparently normal lifespan, *TNNI3K* transgenic animals did not show any signs of cardiac pathology by echocardiography. By contrast, expression of TNNI3K in the context of the *Csq* transgenic sensitizer resulted in profoundly premature death (Figure 5). Of the four possible genotypes from a cross between *Csq* (sensitizer) and *TNNI3K* (modifier) transgenic lines, only the double transgenic mice showed a decrease in survival (p<0.00001). The observed survival differences were profound. All other genotypes survived on average at least 150 days, but all animals expressing both *Csq* and *TNNI3K* died within 21 days. This extreme premature death phenotype resembled that which we had previously observed when attempting to introgress the *Csq* transgene into the B6 background, which exhibits robust levels of endogenous mouse TNNI3K protein [5]. Starting with the sensitizer in the DBA background [4], we were unable to move the *Csq* transgene beyond the second generation, as N2 animals died within 40 days, precluding further backcrosses with B6 mice [5].

We next determined whether *natural* levels of the *murine* TNNI3K protein would also exhibit disease-accelerating effects. This was investigated by crossing the congenic mice harboring the AKR allele at *Hrtfm2* with mice containing the *Csq* transgene, both held for many generations in the DBA background. The progeny from this cross would harbor a only single AKR allele of *Tnni3k*, the appropriate genotype for *Hrtfm2* which exhibited dominant effects in the original mapping crosses [5],[7]. When the congenic mice DBA.AKR-*Hrtfm2*, were crossed with the *Csq* sensitized mice, the *Csq^tg^* offspring with even only a single AKR *Hrtfm2* allele showed a decrease in survival (on average 107 days) compared to those with two DBA *Hrtfm2* alleles (more than 150 days, Figure 5). The *Csq*-sensitized *Hrtfm2* congenic line also survived longer than *Csq*-sensitized F1(DBA/B6) animals, which survived on average to only 50 days [5]. In the original mapping cross, *Hrtfm2* contributed approximately 30% of the genetic variance towards the survival trait [5]. Thus, as expected, the isolated B6/AKR *Hrtfm2* allele contributed a robust but only partial effect on reduced survival when compared to the F1 animals, as other modifier loci had been crossed out of the congenic line.

**Figure 6:**
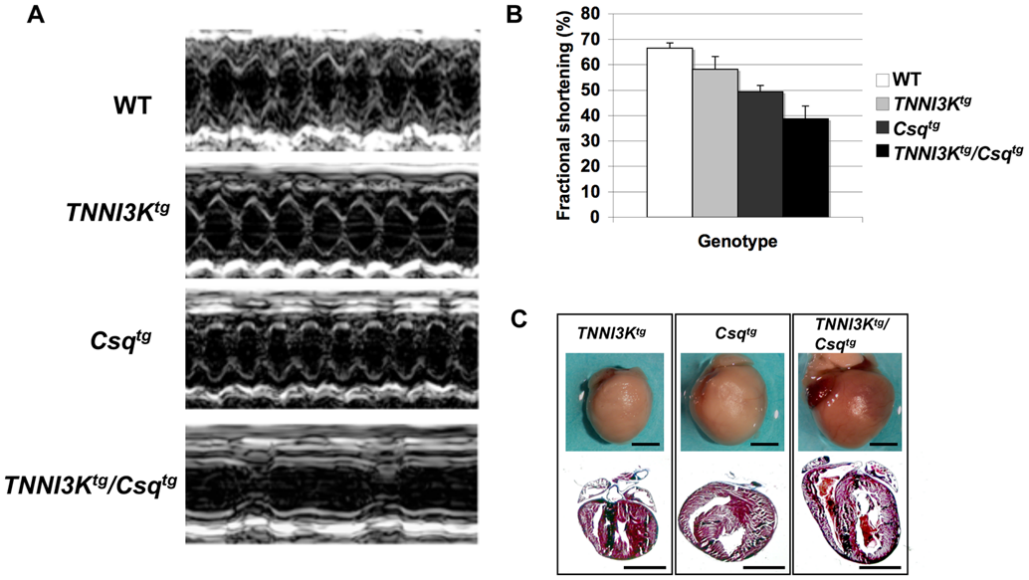
TNNI3K expression leads to severely impaired cardiac function in the *Csq^tg^* transgenic model of cardiomyopathy. M-mode echocardiograms were performed on 14-day-old mice from a cross between *TNNI3K^tg^* and *Csq^tg^* transgenic animals. (A) Representative echocardiograms showed that the double positive transgenic mice display severe left ventricle systolic dysfunction and chamber dilation (in the bottom). (B) Analysis of the echocardiogram data shows that fractional shortening (FS), which indicates cardiac function, is decreased in double positive transgenic mice compared to other littermates. Data is represented by mean±error of the mean (SEM) for wild type, *TNNI3K^tg^*, *Csq^tg^*, and *TNNI3K^tg^*/*Csq^tg^*. The number of animals in each group is as follows: wild type, n = 12; *TNNI3K^tg^*, n = 8; *Csq^tg^*, n = 14; and *TNNI3K^tg^*/*Csq^tg^*, n = 6. (C) Whole hearts and representative histological sections are shown stained with Masson Trichome. Consistent with echocardiographic data, the *TNNI3K^tg^*/*Csq^tg^*mouse hearts show severe dilation in comparion to *TNNI3K^tg^* and *Csq^tg^* single transgenic animals.

To determine whether the premature death was related to cardiac dysfunction, we performed echocardiography on animals with all four possible genotypes resulting from cross between the *Csq* and *TNNI3K* transgenic lines. Echocardiography was performed at 14 days, the earliest possible age for reproducible echocardiographic data. Due to the extremely accelerated disease course and profound reduction in survival, only six of fourteen double transgenic mice survived to their scheduled 14-day echocardiogram. Fractional shortening in these *TNNI3K^tg^/Csq^tg^* mice was significantly decreased compared to the other three genotypes of animals (P<0.0232), demonstrating severely abnormal heart function of the double transgenic animal (Figure 6A and 6B, Table S2). Even by 14 days, hearts from the *TNNI3K^tg^/Csq^tg^* mice were larger than those of the other genotypes, and by histological staining showed obvious chamber dilation (Figure 6C). Thus, the double transgenic animals developed dilated cardiomyopathy by 14 days (or earlier) and all mice of this genotype died before this or shortly thereafter due to heart failure. Many of the double transgenic animals displayed bradycardia (a severe slowing of the heart rate), clearly evident in the echocardiograms. This phenotype, while a feature of the natural disease progression in the *Csq* transgenic model, is normally observed only in adult animals just prior to heart failure [5]. Thus, this hallmark of the natural progression of the *Csq* transgenic model is also greatly accelerated with overexpression of TNNI3K.

**Figure 7:**
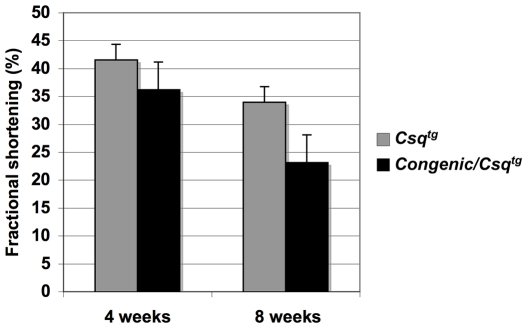
One copy of the AKR allele at *Hrtfm2* leads to an acceleration of cardiomyopathy in the *Csq^tg^* transgenic model of cardiomyopathy. Echocardiographic analysis showed decreased fractional shortening in *Csq^tg^* transgenic mice on the congenic background (DBA. AKR-*Hrtfm2*) at 4 and 8 weeks of age, compared to their littermates with DBA *Hrtfm2* alleles (p = 0.263 and 0.016). From 4 to 8 weeks, the fractional shortening was decreased 36% in *Csq^tg^*/Congenic mice (p = 0.0004), and 18% in *Csq^tg^*/DBA mice (p = 0.186). The number of animals in each group was as follows: *Csq^tg^*/DBA, 4 weeks, n = 7; 8 weeks, n = 7; *Csq^tg^*/Congenic, 4 weeks, n = 9; 8 weeks, n = 6. Data is represented by mean±SEM.

We also investigated the echocardiographic parameters of the DBA.AKR-*Hrtfm2/Csq^tg^* (see Figure 5). Echocardiography performed on these mice at 4 and 8 weeks of age showed decreased fractional shortening in DBA.AKR-*Hrtfm2/Csq^tg^* mice compared to the DBA/*Csq^tg^* littermates, indicating more severe level of cardiomyopathy (Figure 7, Table S3). Furthermore, from age 4 to 8 weeks, percent fractional shortening decreased more rapidly in DBA.AKR-*Hrtfm2/Csq^tg^* mice (36%, p = 0.0004) than the littermates (18%, p = 0.186), suggesting that the presence of even a single AKR-*Hrtfm2* locus (essentially half the normal AKR/B6 level of TNNI3K expression) can accelerate the progression of the *Csq*-induced cardiomyopathy.

**Figure 8:**
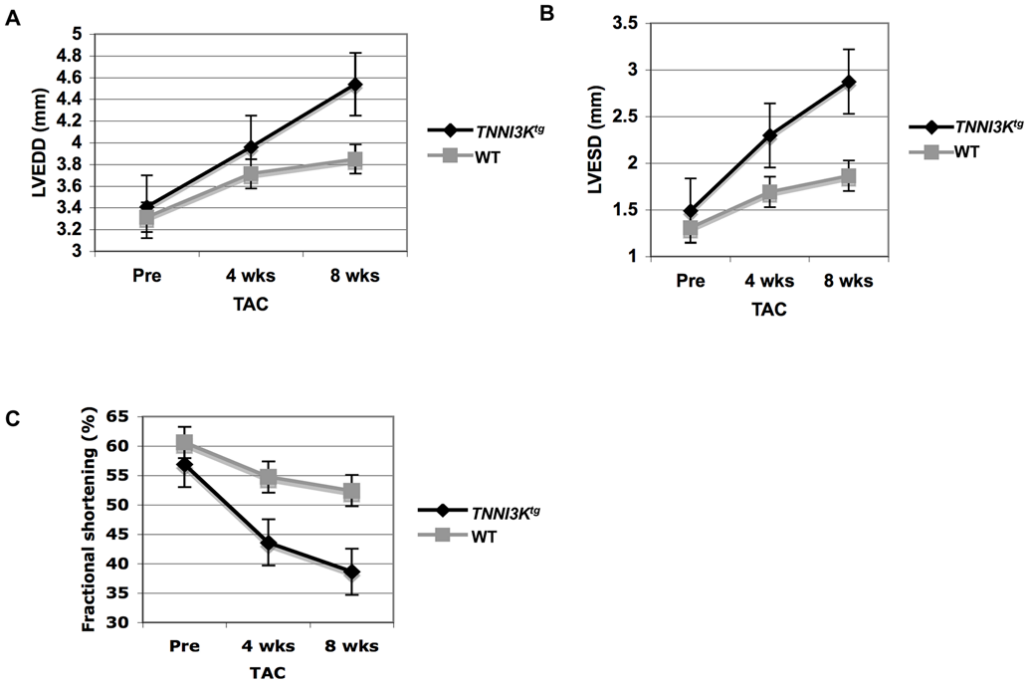
TNNI3K expression leads to systolic dysfunction in a surgically induced model of cardiomyopathy. Echocardiography was performed prior to transverse aortic constriction (TAC) and at 4- and 8-weeks post TAC surgery. (A) Left-ventricular end diastolic diameter (LVEDD), (B) left-ventricular end systolic diameter (LVESD), and (C) FS were compared between *TNNI3K^tg^* mice (n = 11) and wild-type littermates (n = 13) at 4 and 8 weeks post-TAC. LVEDDs were significantly higher in *TNNI3K^tg^* mice at 4 and 8 weeks, but were not statistically different prior to surgery. Similarly, fractional shortening was significantly decreased in *TNNI3K^tg^* mice at both 4 and 8 weeks following surgery. Data is represented by mean±SEM.

We next investigated whether TNNI3K expression would exhibit a disease accelerating effect in a model of cardiomyopathy that was unrelated to Calsequestrin over-expression. Transverse aortic constriction (TAC) induces left ventricular hypertrophy in response to pressure overload [13]. We performed TAC on *TNNI3K* transgenic animals and wild-type littermate controls. Cardiac function was analyzed by echocardiography at 4 and 8 weeks following TAC surgery. At 4 and 8 weeks post-surgery, the transgene-positive mice showed greater diastolic and systolic dysfunction (increased left-ventricular end diastolic diameter (LVEDD) and left-ventricular end systolic diameter (LVESD)), and significantly reduced fractional shortening compare to the control mice (Figure 8A–8C, Table S4). This confirmed that TNNI3K expression has a detrimental effect on heart function outside the context of the transgenic *Csq* sensitizer.

## Discussion

We had previously mapped 7 loci (*Hrtfm1-7*) that modify heart disease progression using the *Calsequestrin* (*Csq*) transgenic mouse model [5]–[7]. Here we report *Tnni3k* (*cardiac Troponin I-interacting kinase*) as the gene underlying *Hrtfm2*. We have shown that the murine *Tnni3k* locus harbors an ancestral SNP in intron 19 that activates a cryptic splice site, generating an aberrant transcript that undergoes NMD, leading to drastically reduced message levels and an apparent absence of TNNI3K protein. In DBA and other inbred mouse strains sharing the same haplotype at *Tnni3k*, drastically reduced levels of TNNI3K protein have no obvious effect on normal development or physiology, suggesting that any trace amounts of protein that remain are sufficient for its normal function, or that the lack of protein is compensated by functional redundancy of another gene. *In vivo* transgenic and congenic mouse lines confirm that TNNI3K levels are a significant determinant of the rate of disease progression and outcome, since expression of this protein accelerates disease progression in two independent and unrelated models of cardiomyopathy. However, we did not observe a simple, linear relationship between the level of TNNI3K transgenic overexpression and the strength of the modifying effect. In these models, the levels of overexpression in the transgenic lines may have crossed the threshold required for maximal phenotypic effects.

The modifying effects of TNNI3K expression were not dependent on the allele at the nonsynonymous coding SNP (rs30712233, T659I). DBA and most inbred mouse strains encode Threonine at this position. Most other species also encode Threonine at the homologous position. By contrast, B6 and AKR inbred strains encode the Isoleucine variant. The human *TNNI3K* transgene employed in the validation experiments coincidentally encodes the highly conserved Threonine variant. The modifying effects of the human transgene carrying the conserved variant (695T) directly parallel, and are even stronger, than those observed using the congenic line containing the B6/AKR variant (695I). Thus, robust phenotype modifying effects were observed independent of the murine *Tnni3k* coding variant. Nonetheless, these experiments did not address whether the 695I polymorphism also alters TNNI3K protein function.

TNNI3K was identified as a cardiac-specific protein kinase that interacted with cardiac Troponin I (cTnI) in the yeast-two hybrid interaction assay [8], however, cTnI has not been established as a phosphorylation target. TNNI3K protein contains seven ankyrin repeats in the N-terminus followed by a dual-specificity protein kinase domain and a short C-terminal serine-rich domain. The overall domain structure of TNNI3K resembles that of Integrin-linked Kinase (ILK). ILK mediates communication from the cellular matrix to intracellular signaling molecules such as PKB and GSK3β, and plays important roles in cardiac growth, contractility and repair [14],[15]. Sequence and structural homology might imply similar functions for TNNI3K. A yeast two-hybrid interaction screen with a C-terminal fragment of TNNI3K identified several additional sarcomeric proteins as putative binding partners such as cardiac α-actin and myosin binding protein C [8]. These studies suggest that TNNI3K might modulate sarcomere function through interactions with key components of the sarcomeric complex. However, to date, none of these proteins has been validated as a phosphorylation target of TNNI3K in cardiomyocytes, and the *in vivo* function of TNNI3K remains unknown.

Recently, expression of TNNI3K was shown to be protective in a different cardiomyopathic disease context [16],[17]. In a murine model of cardiac ischemia, intramyocardial transplantation of *Tnni3k*-overexpressing P19CL6 cells promoted cardiomyogenesis and improved cardiac function. We note that P19CL6 cells were originally derived from the C3H lineage of mice [18], which share with DBA, the “null” haplotype for *Tnni3k* (see Figure 1). Thus, the resulting phenotype may have been due to the restoration of *Tnni3k* expression in otherwise null cells, rather than to overt overexpression of the gene. A locus for susceptibility to coxsackievirus B3-induced myocarditis maps to a locus on distal mouse chromosome 3 (*Vms1*, viral myocarditis susceptibility locus) that virtually overlaps *Hrtfm2* and which includes *Tnni3k* in its confidence interval [17]. In this disease model, inbred strain C57BL/10 provides the protective allele at the locus, and strain A/J provides the susceptibility allele. Assuming that *Tnni3k* also underlies the *Vms1* locus, viral-induced myocarditis might represent another disease context where expression of TNNI3K is protective. These combined data suggest that expression of TNNI3K may be detrimental in certain pathological conditions such as pressure overload or aberrant sarcomeric calcium regulation, but protective in other disease contexts. In either scenario, TNNI3K appears to play a critical role in modulating disease progression and outcome in heart disease.

Since protein kinases are critical cell cycle regulators, kinase inhibitors have become a major avenue for the development of novel cancer therapeutics. TNNI3K may be an ideal candidate for the development of small molecule kinase inhibitors for categories of heart disease where TNNI3K expression is detrimental. In these cases, selective inhibition of TNNI3K would be particularly useful as it might slow disease progression, which may prove beneficial in treating individuals with rapidly progressing disease. In other scenarios of disease, augmentation of TNNI3K activity or protein levels may instead prove beneficial. Further investigation of TNNI3K function in these and other cardiomyopathic mouse models will lead to increased understanding of its role in both normal and pathological contexts, and may provide a novel target for therapy for heart disease.

## Methods

### Animal care and handling

All mice were handled according to approved protocol and animal welfare regulations of the Institutional Review Board at Duke University Medical Center. All inbred mouse strains used in the course of this study were obtained from Jackson Laboratory (Bar Harbor, ME). Transgenic mice overexpressing *Csq* [3] were maintained on a DBA/2J genetic background.

### DBA.AKR-*Hrtfm2* congenic mouse

Through repeated backcrossing to DBA/2J, a congenic mouse was created which retains AKR genomic DNA at the *Hrtfm2* locus in the DBA genetic background. At generation N2, breeders were selected which were heterozygous at *Hrtfm2* and homozygous DBA at the other mapped modifier loci [7]. Genome-wide SNP genotyping was carried out using the Mouse MD linkage panel with 1449 SNPs (Illumina, San Diego, CA). By generation N6, the animals were homozygous for DBA alleles throughout the genome and only showed heterozygosity for an approximately 20 Mb interval on chromosome 3, the region containing *Hrtfm2*. Once we had reached the generation N10 backcross, the DBA.AKR-Hrtfm2 mouse was maintained by intercross.

### Mouse RNA isolation, microarray analysis, and qRT-PCR

Whole hearts removed from age- and sex-matched wild type animals from each of the three primary strains (B6, DBA, AKR) were used to examine RNA transcript levels. Total RNA was isolated using the RNeasy Kit (Qiagen, Valencia, CA). Microarray analysis was done on an Affymetrix Mouse probe set (Mouse 430 2.0 Array, Affymetrix, Santa Clara, CA). Analysis was done using GeneSpring GX 7.3 Expression Analysis (Agilent Technologies, Santa Clara, CA). For the TaqMan expression analysis, total RNA was extracted from whole mouse hearts using TRIzol reagent (Invitrogen, Carlsbad, CA). cDNA was synthesized from 1 µg total RNA using the High Capacity cDNA Archive Kit (Applied Biosystems, Foster City, CA) and used as the template for qRT-PCR. *Tnni3k* cDNA was amplified using the predesigned gene expression assay (TaqMan, ABI, assay ID: Mm01318633_m1). Beta-actin (Actb) was used as the endogenous control (TaqMan, ABI, catalogue number 4352341E). All amplifications were carried out in triplicate on an ABI Prism 7000 Real Time PCR system and analyzed with ABI software. All statistical analyses were done using an unpaired, two-tailed T-test.

### Analysis of TNNI3K protein expression

Whole heart protein lysates were prepared using flash-frozen heart tissue resuspended in lysis buffer with protease inhibitors. Lysates were analyzed by SDS-PAGE and Western blot performed with standard methods. A polyclonal peptide antiserum was developed to the C-terminal 14 amino acids of mouse TNNI3K protein (LHSRRNSGSFEDGN). Antiserum from 2 rabbits was purified on a Protein A column (GenScript, Piscataway, NJ). TNNI3K antibody was used at a 1∶1000 dilution in TBST with 5% dry milk. Secondary anti-rabbit antibody conjugated to HRP followed by incubation with Pierce SuperSignal West Pico Chemiluminescant Substrate (Thermo Fisher Scientific, Rockford, IL) and exposure to X-OMAT film (Kodak) to visualize protein bands. Western blot analysis was used to confirm specificity of the antibody. As predicted, the mTNNI3K antibody detects a 90 kDa protein from lysates prepared from 293T cells transiently transfected with a full length *Tnni3k* expression vector and in protein lysates from wild-type mouse hearts.

### Fluorescent RT-PCR assay

cDNAs were subjected to qRT-PCR using primers designed to detect either a 116 bp or a 120 bp cDNA PCR product. The forward primer was targeted 25 bp upstream of the predicted 4 base insertion and was fluorescently labeled: 5′-6FAM-AGATTTCTGCAGTCCCTGGAT-3′ while the unlabeled reverse primer was targeted 48 bp downstream of the predicted 4 base insertion with the sequence: 5′-AAGACATCAGCCTTGATGGTG-3′. Accumulation of both fragments was quantified using the GeneMapper analysis program on the ABI Prism 3730 DNA Sequencer (Applied Biosystems). Ratios of properly spliced and mis-spliced products were calculated based on relative amplification of both cDNA products.

### Cloning of m*Tnni3k* splicing constructs, cell culture, and transfection

To create the *Tnni3k* genomic splicing constructs, DBA genomic DNA and B6 BAC clone RP23-180023 were used as templates to generate genomic 4 kb fragments that included part of intron 17, exon 18, intron 18, exon 19, intron 19, exon 20 and part of intron 20. The sequence of the forward PCR primer was 5′-ACTTACTTATGTGCTTCTCTTAGTTATGTGC-3′; the reverse primer was 5′-GGATTTAAACATAGGTGTGTACCTAATTGT-3′. PCR products were sub-cloned into pSPL3 (Invitrogen). Clones were verified by direct sequencing. Human embryonic kidney HEK293T (293T) cells (ATCC, Manassas, VA) were maintained in Dulbecco's Modified Eagle's Medium (DMEM, Gibco) containing 10% fetal bovine serum at 37°C in 5% CO_2_. Cells were grown on 35 mm^2^ plates and transfected with 1 µg plasmid DNA using FuGene reagent (Roche, Indianapolis, IN) according to the manufacturer's protocol. RNA was extracted with TRIzol (Invitrogen) 24 hr post-transfection and RT-PCR was carried out using standard methods.

### *In vitro* splicing assay

HEK293T cells were grown to approximately 80% confluence in 6-well plates, then transfected using with 1 µg of DBA- or B6-pSPL3 plasmid mixed with FuGene reagent. All transfections were performed in triplicate. Total RNA was extracted with TRIzol 20 hr post-transfection. RT-PCR was carried out using standard methods. Ratios of properly spliced and aberrantly spliced products for the Tnni3k construct were determined by the fluorescent RT-PCR assay described above.

### Site-directed mutagenesis

A single base was changed at rs49812611 (IVS19+9), in the DBA-pSPL3 construct (G→A) and the B6-pSPL3 construct (A→G) using the QuikChange Site-Directed Mutagenesis Kit (Stratagene, LaJolla, CA) with PfuTurbo proofreading DNA polymerase. All clones were sequenced to verify proper incorporation of the SNP.

### Culture of cardiomyocytes and NMD blocking experiments

HL-1 cardiomyocytes [11] were cultured in Claycomb Medium (SAFC Laboratories, Lenexa, KS) supplemented with Fetal Bovine Serum at 10%, 2 mM L-Glutamine, 100 mg/ml Penicillin/Streptomycin, and 100 mM fungizone. Cells were cultured at 37°C with 5% CO_2_. Although the HL-1 cardiomyocytes were derived from a heart isolated from a mixed B6-DBA mouse [11] direct sequencing of genomic DNA from the cell line showed that it is homozygous for DBA alleles at the *Tnni3k* locus. HL-1 cells were treated with 5.7×10^−2^ mM cycloheximide or 3.3×10^−2^ mM emetine. Each treatment was performed in triplicate and RNA was isolated from cells 24 hours post treatment. RT-PCR was performed on RNA isolated from cells treated with NMD blocking drugs and untreated controls. Ratios of properly spliced and aberrantly spliced products were measured using the fluorescent RT-PCR splicing assay as described above. Total transcript levels were determined using the *Tnni3k* TaqMan assay described above.

### Creation and testing of a *TNNI3K* transgenic mouse

A full-length 2.5 kb human *TNNI3K* cDNA was amplified from normal human heart RNA following RT-PCR and cloned into a vector downstream of the murine α-myosin heavy chain (αMHC) promoter. An artificial minx intron was inserted upstream of the *TNNI3K* start codon. The construct was linearized and an 8 kb fragment containing the αMHC promoter, cDNA and SV40 polyadenylation sequence was purified and used for microinjection. B6SJLF1/J blastocysts were injected with the linearized transgene and subsequently implanted into surrogate mice. The resulting founder animals were genotyped for presence of the *TNNI3K* transgene using a 5′ primer in the αMHC promoter and a 3′ primer in the *TNNI3K* transgene. Three transgenic lines were chosen for backcrossing to the DBA strain. Western blot analysis of heart lysates with a polyclonal antibody (Bethyl Laboratories, Montgomery, TX) raised against a human C-terminal TNNI3K peptide (FHSCRNSSSFEDSS) confirmed similar levels of expression of the *TNNI3K* transgene in each line (Figure S1). This was repeated for several generations of backcrossing to DBA. Southern blot analysis of DNA from founder animals and subsequent generations (N2–N3) indicated that two founder lines carried 10–20 copies of the transgene while the third line appeared to have >100 copies. qRT-PCR with SYBRgreen (Invitrogen) was performed on heart cDNA from several transgenic mice to determine the relative expression difference between endogenous mouse *Tnni3k* and transgenic human *TNNI3K* expression.

### M-mode echocardiography

Transthoracic two-dimensional M-mode echocardiography was performed between 12 and 18 weeks of age in conscious mice using either a Vevo 770 echocardiograph (Visual Sonics, Toronto, Canada) or an HDI 5000 echocardiograph with a 15-MHz frequency probe (Phillips Electronics, Bothell, WA). Measurements of cardiac function include heart rate, posterior and septal wall thickness, left-ventricular end diastolic diameter (LVEDD) and left-ventricular end systolic diameter (LVESD). Fractional shortening (FS) was calculated with the formula: FS = (LVEDD−LVESD)/LVEDD, as previously described [4].

### Histology

Hearts were fixed in 10% neutral buffered formalin, dehydrated in 75%, 90% and 100% ethanol, and embedded in paraffin; sections 5 mm in thickness were cut and then stained with Masson's trichrome stain.

### Transverse aortic constriction

Mice were anesthetized with a mixture of ketamine (100 mg/kg) and xylazine (2.5 mg/kg), and transverse aortic constriction (TAC) was performed as previously described [13]. TAC was performed on 14 *TNNI3K* transgene-positive animals and 14 transgene-negative (wild-type) littermates at 10 weeks of age. One of the transgene-negative controls and three transgene-positive animals died following surgery, which is a normal complication of this procedure. The remaining 24 mice were then analyzed by echocardiography (as described above), at 4 and 8 weeks following the surgery.

## Supporting Information

Figure S1 Calsequestrin and TNNI3K protein expression in transgenic lines. (A) Calsequestrin protein expression detected in *Csq^tg^* and *Csq^tg^*; *TNNI3K^tg^* mice heart lysates using anti-CSQ polycolonal antibody. A representative result is shown. (B) Stable expression of human TNNI3K protein in two independent transgenic lines. TNNI3K expression was detected in mice heart lysates from both *TNNI3K^tg^* lines using anti-TNNI3K polycolonal antibody. A representative result is shown from N7 backcross animals for both transgenic lines.
(0.49 MB TIF)

Table S1 Coding and representative non-coding polymorphic SNPs from the mouse *Tnni3k* genomic region show two distinct haplotype groups. The two SNP haplotypes correlate with *Tnni3k* transcript levels. Group 1 (DBA, C3H, and BALB/c) show low levels of *Tnni3k* while group 2 (B6, AKR, and 129Sv) show high levels of *Tnni3k*.
(0.11 MB DOC)

Table S2 M-mode echocardiograms of 14-day-old mice from a cross between *TNNI3K^tg^* and *Csq^tg^* transgenic animals. Measurements of cardiac function included left-ventricular end diastolic diameter (LVEDD), left-ventricular end systolic diameter (LVESD), posterior (PW) and septal (IVSW) wall thickness, ejection time (ET), and heart rate (HR). Heart weight (HW) and body weight (BW) were measured and the ratio of heart weight to body weight was determined. Data is shown as mean±sd.
(0.07 MB DOC)

Table S3 M-mode echocardiograms of *Csq^tg^* transgenic mice on the congenic background (DBA.AKR-*Hrtfm2*) at 4 and 8 weeks ages, compared to their littermates with DBA *Hrtfm2* alleles. Measurements of cardiac function included left-ventricular end diastolic diameter (LVEDD), left-ventricular end systolic diameter (LVESD), posterior (PW) and septal (IVSW) wall thickness, ejection time (ET), and heart rate (HR). Data is shown as mean±sd.
(0.07 MB DOC)

Table S4 M-mode echocardiograms for a surgically induced model of cardiomyopathy. Echocardiography was performed prior to transverse aortic constriction (TAC) and at 4- and 8-weeks post TAC surgery on *TNNI3K^tg^* mice and wild type littermates. Measurements of cardiac function included left-ventricular end diastolic diameter (LVEDD), left-ventricular end systolic diameter (LVESD), posterior (PW) and septal (IVSW) wall thickness, ejection time (ET), and heart rate (HR). Data is shown as mean±sd.
(0.09 MB DOC)
