# Supplementary figures and images for: Tnni3k Modifies Disease Progression in Murine Models of Cardiomyopathy
Source: PLoS Genet. 2009 Sep 18;5(9):e1000647. doi: 10.1371/journal.pgen.1000647 (PMC2731170; doi:10.1371/journal.pgen.1000647)

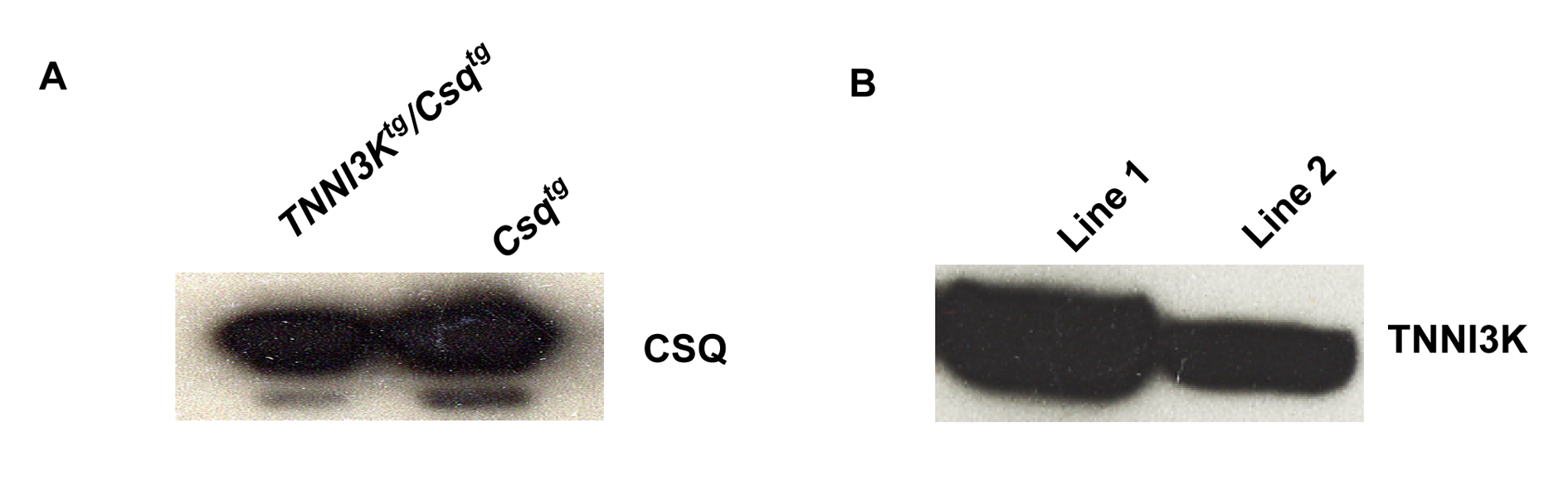

Supplement: Figure S1 — Calsequestrin and TNNI3K protein expression in transgenic lines. (A) Calsequestrin protein expression detected in Csqtg and Csqtg; TNNI3Ktg mice heart lysates using anti-CSQ polycolonal antibody. A representative result is shown. (B) Stable expression of human TNNI3K protein in two independent transgenic lines. TNNI3K expression was detected in mice heart lysates from both TNNI3Ktg lines using anti-TNNI3K polycolonal antibody. A representative result is shown from N7 backcross animals for both transgenic lines. (0.49 MB TIF) [file pgen.1000647.s001.tif]
