# Supplementary material for: Tnni3k Modifies Disease Progression in Murine Models of Cardiomyopathy
Source: PLoS Genet. 2009 Sep 18;5(9):e1000647. doi: 10.1371/journal.pgen.1000647 (PMC2731170; doi:10.1371/journal.pgen.1000647)
Supplement: Table S1 — Coding and representative non-coding polymorphic SNPs from the mouse Tnni3k genomic region show two distinct haplotype groups. The two SNP haplotypes correlate with Tnni3k transcript levels. Group 1 (DBA, C3H, and BALB/c) show low levels of Tnni3k while group 2 (B6, AKR, and 129Sv) show high levels of Tnni3k. (0.11 MB DOC) [file pgen.1000647.s002.doc]

Table S1. Coding and representative non-coding polymorphic SNPs from the mouse *Tnni3k* genomic region show two distinct haplotype groups.
