# Supplementary material for: Tnni3k Modifies Disease Progression in Murine Models of Cardiomyopathy
Source: PLoS Genet. 2009 Sep 18;5(9):e1000647. doi: 10.1371/journal.pgen.1000647 (PMC2731170; doi:10.1371/journal.pgen.1000647)
Supplement: Table S2 — M-mode echocardiograms of 14-day-old mice from a cross between TNNI3Ktg and Csqtg transgenic animals. Measurements of cardiac function included left-ventricular end diastolic diameter (LVEDD), left-ventricular end systolic diameter (LVESD), posterior (PW) and septal (IVSW) wall thickness, ejection time (ET), and heart rate (HR). Heart weight (HW) and body weight (BW) were measured and the ratio of heart weight to body weight was determined. Data is shown as mean±sd. (0.07 MB DOC) [file pgen.1000647.s003.doc]

Table S2. M-mode echocardiograms analysis of 14-day-old mice from a cross between *TNNI3K^tg^* and *Csq^tg^* transgenic animals.
