# Supplementary material for: Tnni3k Modifies Disease Progression in Murine Models of Cardiomyopathy
Source: PLoS Genet. 2009 Sep 18;5(9):e1000647. doi: 10.1371/journal.pgen.1000647 (PMC2731170; doi:10.1371/journal.pgen.1000647)
Supplement: Table S3 — M-mode echocardiograms of Csqtg transgenic mice on the congenic background (DBA.AKR-Hrtfm2) at 4 and 8 weeks ages, compared to their littermates with DBA Hrtfm2 alleles. Measurements of cardiac function included left-ventricular end diastolic diameter (LVEDD), left-ventricular end systolic diameter (LVESD), posterior (PW) and septal (IVSW) wall thickness, ejection time (ET), and heart rate (HR). Data is shown as mean±sd. (0.07 MB DOC) [file pgen.1000647.s004.doc]

Table S3. M-mode echocardiograms analysis of *Csq^tg^* transgenic mice on the congenic background (DBA. AKR-*Hrtfm2*) at 4 and 8 weeks ages, compared to their littermates with DBA *Hrtfm2* alleles.
