# Supplementary material for: Tnni3k Modifies Disease Progression in Murine Models of Cardiomyopathy
Source: PLoS Genet. 2009 Sep 18;5(9):e1000647. doi: 10.1371/journal.pgen.1000647 (PMC2731170; doi:10.1371/journal.pgen.1000647)
Supplement: Table S4 — M-mode echocardiograms for a surgically induced model of cardiomyopathy. Echocardiography was performed prior to transverse aortic constriction (TAC) and at 4- and 8-weeks post TAC surgery on TNNI3Ktg mice and wild type littermates. Measurements of cardiac function included left-ventricular end diastolic diameter (LVEDD), left-ventricular end systolic diameter (LVESD), posterior (PW) and septal (IVSW) wall thickness, ejection time (ET), and heart rate (HR). Data is shown as mean±sd. (0.09 MB DOC) [file pgen.1000647.s005.doc]

Table S4. M-mode echocardiograms analysis of surgically-induced model of cardiomyopathy.
